# Urinary TIMP2 and IGFBP7 Identifies High Risk Patients of Short-Term Progression from Mild and Moderate to Severe Acute Kidney Injury during Septic Shock: A Prospective Cohort Study

**DOI:** 10.1155/2019/3471215

**Published:** 2019-04-01

**Authors:** Julien Maizel, Delphine Daubin, Ly Van Vong, Dimitri Titeca-Beauport, Morgane Wetzstein, Loay Kontar, Michel Slama, Kada Klouche, Christophe Vinsonneau

**Affiliations:** ^1^Medical ICU and EA7517, Amiens University Hospital, Amiens, France; ^2^Medical ICU, Montpellier University Hospital, Montpellier, France; ^3^Medico-Surgical ICU, Melun General Hospital, Melun, France

## Abstract

**Background:**

To examine whether the new urinary biomarkers TIMP2 and IGFBP7 can predict progression within 24 hours and 72 hours from mild and moderate (KDIGO 1 or 2) to severe (KDIGO 3) AKI in patients with septic shock.

**Methods:**

A prospective, multicenter observational study performed in three French ICUs. The urinary biomarkers TIMP2∗IGFBP7 were analyzed at the early phase (<6 hours) of patients admitted for septic shock with mild and moderate AKI.

**Results:**

Among the 112 patients included, 45 (40%) progressed to the KDIGO 3 level 24 hours after inclusion (KDIGO 3 H24) and 47 (42%) 72 hours after inclusion (KDIGO 3 H72). The median urinary TIMP2∗IGFBP7 at inclusion (baseline) were higher in the KDIGO 3 group than in the KDIGO<3 group at H24 and H72. All covariates with a *p* value < 0.1 in the univariate analysis were included in stepwise multiple logistic regression models to identify factors independently associated with the risk of KDIGO 3 at H24 and H72. TIMP2∗IGFBP7 remained independently associated with KDIGO 3 at H24 and H72. Baseline posology of norepinephrine, baseline urine output, and baseline serum creatinine remained also significantly associated with progression to KDIGO 3 at H24. Baseline TIMP2∗IGFBP7 and baseline urinary output had the best AUC ROC. A baseline TIMP2∗IGFBP7 > 2.0 (ng/ml)^2^/1,000 identified the population at high risk of KDIGO 3 H24 (relative risk 4.19 (1.7-10.4)) with a sensitivity of 76% (60-87) and a specificity of 81% (69-89). But the diagnostic performance at H72 of baseline TIMP2∗IGFBP7 was poor (AUC: 0.69 (0.59-0.77)).

**Conclusion:**

The urinary TIMP2∗IGFBP7 concentration and the urine output at the early phase of septic shock are independent factors to identify the population at high risk of progression from mild and moderate to severe AKI over the next 24 but not 72 hours. A TIMP2∗IGFBP7 concentration > 2.0 (ng/ml)^2^/1,000 quadruples the risk of KDIGO 3 AKI within 24 hours. This trial is registered with (NCT03547414).

## 1. Background

Septic shock is one of the leading causes of death in patients admitted to the intensive care unit (ICU) [1]. Acute kidney injury (AKI) occurs in almost 50% of septic patients and is associated with significant mortality [2]. The definition and staging of AKI have been standardized. The Kidney Disease: Improving Global Outcomes (KDIGO) consensus classification defines three stages of AKI (AKIN classification) based on the patient's urine output and serum creatinine [3]. The survival rate of ICU patients decreases with incremental staging of this classification [4]. Patients who will progress to severe AKI (KDIGO stage 3) and who present the highest risk of death can be poorly discriminated from patients who will remain below stage 3 and eventually return to normal kidney function (transient AKI) [5]. Renal biomarkers are unable to accurately identify those patients who will progress to severe AKI (KDIGO 3) [5].

A new urine test, the NephroCheck™, has been validated [6]. It corresponds to the product of the urinary concentrations of 2 markers of renal tubule injury (TIMP2 and IGFBP7) associated with a risk of developing AKI KDIGO 2 or 3 within 12 hours. The product of the urinary concentrations of TIMP2 and IGFBP7, two cell cycle arrest proteins, has been shown to predict the development of AKI within 12 to 24 hours [7, 8]. The studies have been performed in unselected critical care patients [9] and in different settings: emergency room [10], postcardiac surgery [11], and septic patients [12]. TIMP2 and IGFBP7 are both released by tubular cells exposed to septic and/or haemodynamic aggression. Although TIMP2∗IGFBP7 can distinguish patients who will subsequently develop AKI, it is unknown whether the baseline urinary concentrations of these biomarkers are also associated with the risk of progression from mild and moderate (KDIGO 1 or 2) to severe (KDIGO 3) AKI. The aim of this study was to determine whether the urinary TIMP2∗IGFBP7 concentration can identify patients with a high risk of progression to KDIGO level 3 in patients with septic shock and AKI KDIGO 1 or 2.

## 2. Methods

This protocol was approved by the Institutional Review Board (IRB North-West II). Patients or their surrogates were informed and could decline to participate at any time, and their decision was recorded in the patient's files.

All patients admitted in the 16-bed medical ICU in Amiens University Hospital (France) between March 2014 and September 2016 and all patients admitted in the 32-bed medical ICU in Montpellier University Hospital (France) and the 22-bed medical and surgical ICU in Melun General Hospital (France) between September 2015 and September 2016 with septic shock according to the bone criteria and AKI KDIGO 1 or 2 within 6 hours after initiation of catecholamines were prospectively included in this study in order to determine the diagnostic value of TIMP2∗IGFBP7 to identify patients at high risk of KDIGO 3. Exclusion criteria were patients without AKI (KDIGO 0), anuria, severe AKI (KDIGO 3), chronic kidney disease (creatinine clearance < 15 ml/min), decision to withhold treatment, cardiac arrest, age < 18 years, or pregnancy.

Basal serum creatinine was defined as the serum creatinine level measured during the 12 months preceding the onset of septic shock. In the absence of a previous serum creatinine assay, basal serum creatinine was estimated according to the KDIGO guideline [3]. All “baseline” parameters have been recorded at inclusion. Baseline urine output was the volume of urine excreted by hour during the 4 to 6 hours preceding the inclusion.

### 2.1. Measurements

A fresh urine sample was collected on inclusion (maximum 6 hours after starting catecholamines) through the urine collecting tube and frozen at -80°C. At the end of the study, urine samples were thawed and centrifuged as recommended by the manufacturer and the urinary TIMP2∗IGFBP7 concentration was determined using the NephroCheck™ test (Astute Medical Inc., San Diego, CA, USA). The NephroCheck™ test simultaneously measures into the Astute 140™ Meter (a benchtop analyzer) the urinary concentrations of TIMP2 and IGFBP7 on 100 *μ*l of urine mixed with 100 *μ*l of buffer. The result is expressed as a single number corresponding to the product of the TIMP2 and IGFBP7 concentrations. The coefficients of variation (CV) given by the manufacturer for the interassay are comprised of between 8.1% and 11.4% for TIMP2 and between 6.6% and 7.9% for IGFBP7. The CV for the intra-assay are comprised of between 8.0% and 10.7% for TIMP2 and 6.3% and 7.7% for IGFBP7.

### 2.2. Study Endpoints

AKI was categorized according to KDIGO guidelines at baseline, 24 hours, and 72 hours after inclusion [3].

Patients were classified 24 and 72 hours after inclusion according to the progression of the KDIGO classification or death: patients who remained KDIGO<3 and patients who progressed to KDIGO 3 or died.

### 2.3. Statistics

Results are expressed as median (95% confidence interval), and categorical variables are expressed as *n* (%). Comparisons between groups were performed using the Mann-Whitney test or chi-square test, as appropriate.

The performance of the different parameters to identify patients with high risk of deterioration of AKI to KDIGO 3 was tested using multiple logistic regression models including all covariates with a *p* value < 0.1 in the univariate analysis. All significant variables in the first logistic regression model were than retested in a second logistic regression model. Because the diagnostic performance of the various parameters depends on the predictive time frame, analyses were repeated to predict KDIGO 3 at 24 hours and KDIGO 3 or death at 72 hours after inclusion. Nagelkerke pseudo-*R*^2^, a marker of the strength of the final model, is presented. A Nagelkerke pseudo-*R*^2^ close to 1 indicates that the full model reliably predicts the outcome.

The diagnostic value of each parameter independently associated with the progression to KDIGO 3 in the logistic regression models was determined using receiver operating characteristic (ROC) curve analysis. The best parameter was determined by comparing the area under the curve (AUC) using the Hanley-McNeil test at each time point. The best cutoff values to identify patients at high risk of progression to KDIGO 3 at H24 and H72 were determined. Also, we looked at the existing cutoffs for TIMP2∗IGFBP7 (0.3 and 2.0 (ng/ml)^2^/1,000) described previously [9]. Sensitivity, specificity, and negative and positive predictive values were calculated. A *p* value < 0.05 was considered significant. Statistical analysis was performed using MedCalc version 18.6 (MedCalc Software, Mariakerke, Belgium) software.

## 3. Results

### 3.1. Study Endpoints and Population Characteristics

During the study period, 2,800 patients were admitted in the 3 participating centers, including 825 patients with septic shock. One hundred and twelve of these patients were included in the study, while 713 patients were excluded because they presented at least one exclusion criterion: 487 because of the absence of AKI, 175 because of severe AKI (anuria or KDIGO 3 at admission), and the remaining 51 for various reasons (cardiac arrest, chronic renal failure, or withholding of treatment).

**Figure 1:**
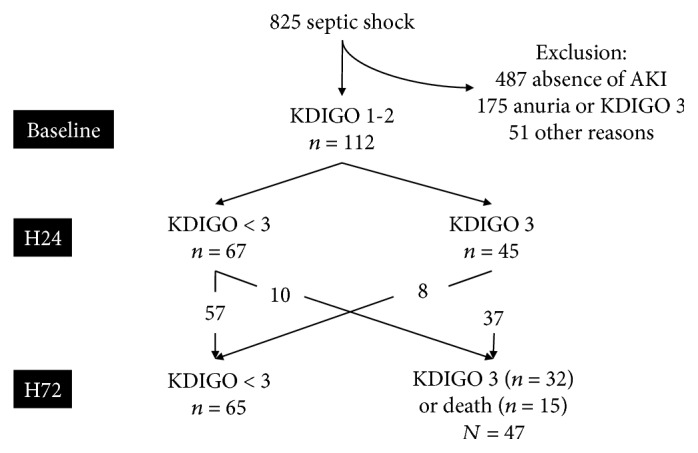
Flow chart.

During the 24 hours following inclusion, 45 patients developed KDIGO 3 AKI (diagnostics based on creatinine elevation in 14 patients, low urinary output in 9 patients, and both criteria in 22 patients) and 67 remained below KDIGO 3. At H72, 32 patients were KDIGO 3, 65 were KDIGO<3, and 15 patients had died (Figure 1).

### 3.2. Identification of Patients at High Risk of Severe AKI at H24

**Figure 2:**
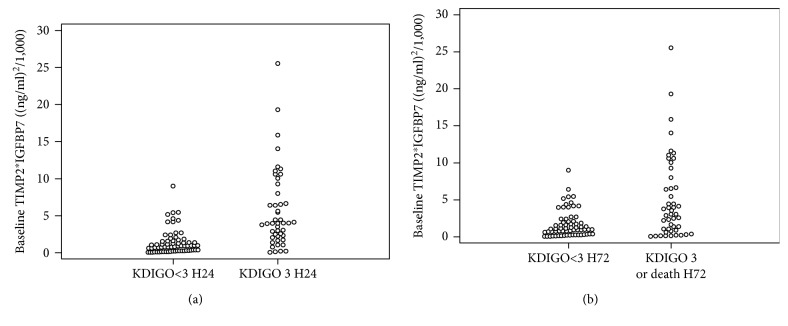
Individual baseline TIMP2∗IGFBP7 in patients who developed and who did not develop KDIGO 3 AKI at H24 (a) and H72 (b).

Patients with a diagnosis of KDIGO 3 AKI 24 hours following inclusion (KDIGO 3 H24) presented at inclusion a higher posology of norepinephrine, volume of fluid administered, and lactate level (Table 1). The rate of mortality in the KDIGO 3 group of patients was higher than that in the KDIGO<3 patients (28 (62%) vs. 21 (31%), respectively; *p* = 0.002). The KDIGO 3 H24 group had a significantly higher median baseline urinary TIMP2∗IGFBP7 concentration (3.99 (2.17-9.45) (ng/ml)^2^/1,000) compared to the KDIGO<3 H24 group (0.93 (0.25-1.59); *p* = 0.001) (Table 1 and Figure 2). Median baseline urine output and baseline serum creatinine were, respectively, higher and lower in the KDIGO<3 H24 group than in the KDIGO 3 H24 group (Table 1).

In the logistic regression model, only the baseline posology of norepinephrine, baseline TIMP2∗IGFBP7, baseline urine output, and baseline serum creatinine remained significantly associated with progression to KDIGO 3 at H24 (Table 2).

**Figure 3:**
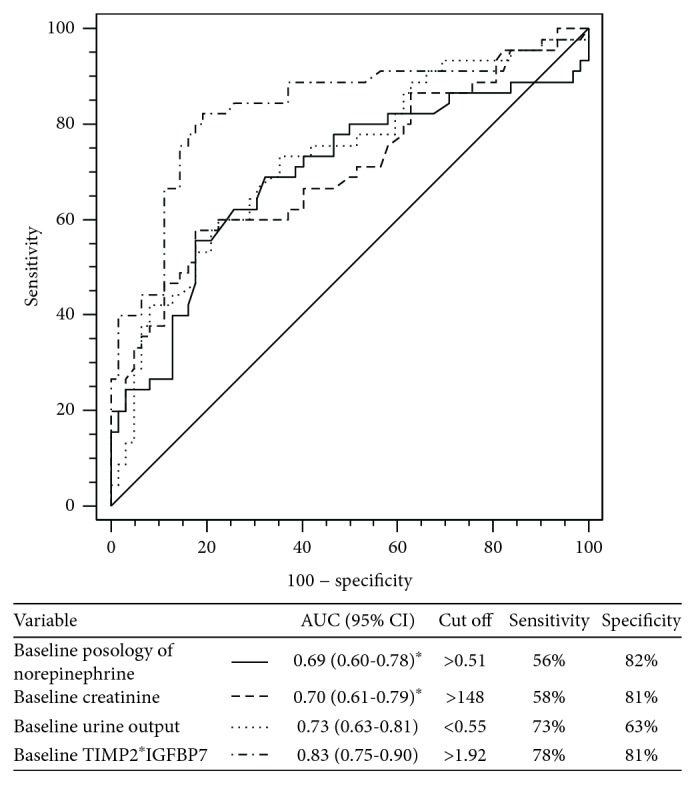
Receiver operating curve for the diagnostic of severe AKI (KDIGO 3) within 24 hours following inclusion. ^∗^*p* < 0.05 vs. baseline TIMP2∗IGFBP7.

The value of urinary TIMP2∗IGFBP7 concentration to identify patients at high risk of KDIGO stage 3 during the 24 hours following inclusion was superior to that of the baseline serum creatinine (AUC: 0.83 (0.75-0.90) vs. 0.70 (0.61-0.79), respectively; *p* = 0.04) and baseline posology of norepinephrine (0.69 (0.60-0.78), *p* = 0.03) but was not significantly different to that of the baseline urine output (0.73 (0.63-0.81); *p* = 0.08) (Figure 3). A TIMP2∗IGFBP7 > 1.92 (ng/ml)^2^/1,000 identified patients who progressed to the KDIGO 3 within 24 hours with a sensitivity of 78% (63-89), a specificity of 81% (69-89), a positive predictive value of 73% (58-84), and a negative predictive value of 84% (73-92). The TIMP2∗IGFBP7 AUC to identify KDIGO 3 were not significantly different between the KDIGO 1 (*n* = 73) and KDIGO 2 (*n* = 39) patients at admission (*p* = 0.09).

**Figure 4:**
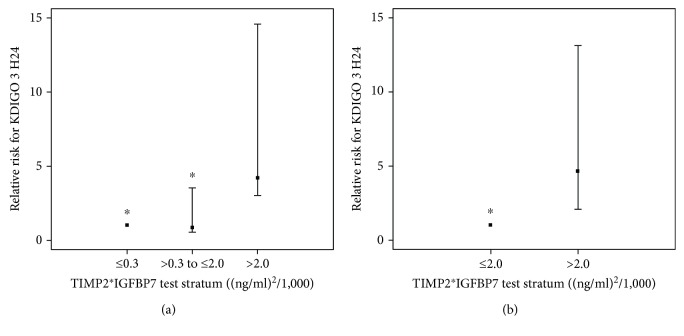
Relative risk of acute kidney injury (AKI) KDIGO 3 level within 24 hours (KDIGO 3 H24) in TIMP2∗IGFBP7 strata. (a) KDIGO 3 H24 risk in the stratum with TIMP2∗IGFBP7 values that are between 0.3 and 2.0 and greater than 2.0 relative to the values less than or equal to 0.3, and (b) KDIGO 3 H24 risk in the stratum with TIMP2∗IGFBP7 values that are greater than 2.0 relative to the values less than or equal to 2.0. Error bars are 95% confidence interval. ^∗^*p* < 0.05 versus >2.0.

The previously published cutoffs for TIMP2∗IGFBP7 to identify critically ill patients at high risk of developing moderate to severe AKI were 0.3 and 2.0 (ng/ml)^2^/1,000 defining the lowest risk to develop an AKI as below 0.3, the intermediate risk as between 0.3 and 2.0, and high risk if higher than 2.0 (9). In our population, a TIMP2∗IGFBP7 < 0.3 identified the group of patients with the lowest risk of developing KDIGO 3 with a sensitivity of 30% (19-42) and specificity of 91% (79-97). A TIMP2^∗^IGFBP7 > 2.0 identified the population at high risk of KDIGO 3 with a sensitivity of 76% (60-87) and a specificity of 81% (69-89). Compared with patients with a TIMP2∗IGFBP7 below 0.3, those with a test score between 0.3 and 2.0 had the same risk for severe AKI (relative risk 0.84 (0.3-2.7, *p* = 0.77)) whereas those with a test score > 2.0 had 4 times the risk for severe AKI (4.19 (1.7-10.4, *p* = 0.002)) (Figure 4).

### 3.3. Identification of Patients at High Risk of Severe AKI at H72

Patients with a diagnosis of KDIGO 3 72 hours following inclusion (KDIGO 3 H72) had a higher posology of norepinephrine, volume of fluid administered, lactate level, and lower mean arterial pressure at inclusion (Table 1). Urine output (0.71 (0.54-0.95) vs. 0.31 (0.2-0.49) ml/kg/h; *p* = 0.001), serum creatinine (114 (106-131) vs. 139 (122-170) *μ*mol/l; *p* = 0.009), and TIMP2∗IGFBP7 (1.03 (0.77-1.51) vs. 3.03 (1.81-5.11); *p* = 0.001) at inclusion were significantly different between the KDIGO<3 H72 and KDIGO 3 H72 groups (Table 1 and Figure 2). In the logistic regression model including the presence of baseline mean arterial pressure, baseline posology of norepinephrine, baseline fluid administered, baseline lactate, baseline TIMP2∗IGFBP7, baseline urine output, and baseline serum creatinine, only the baseline creatinine and baseline TIMP2∗IGFBP7 remained significantly associated with progression to KDIGO 3 at H72 (Table 2). Those two variables were then introduced alone in the regression model, and only TIMP2∗IGFBP7 at baseline remained significantly associated with KDIGO 3 at H72 (Table 2).

The diagnostic performance of baseline creatinine and baseline TIMP2∗IGFBP7 was poor (AUC: 0.64 (0.55-0.73) and 0.69 (0.59-0.77), respectively). A baseline creatinine > 156 *μ*mol/l predicted the KDIGO 3 level 72 hours after inclusion with a sensitivity of 47% (32-62), a specificity of 77% (65-86), a positive predictive value of 59% (42-75), and a negative predictive value of 67% (55-77). A TIMP2∗IGFBP7 > 2.08 predicted the KDIGO 3 level H72 with a sensitivity of 64% (48-77), a specificity of 75% (63-85), a positive predictive value of 65% (54-75), and a negative predictive value of 74% (66-81).

## 4. Discussion

In the early phase of septic shock, the urinary concentration of TIMP2∗IGFBP7 identifies the patients at high risk of progression from mild or moderate to severe AKI during the following 24 hours but not 72 hours. A test score > 2.0 (ng/ml)^2^/1,000 quadrupled the risk of progressing to KDIGO 3.

TIMP2 and IGFBP7 are two proteins released in the urine by renal tubular cells in response to injury. These molecules are released in the case of inflammation and ischaemia of tubular cells in order to block the cell cycle of adjacent tubular cells [13, 14]. Many studies have already demonstrated the relationship between high urinary TIMP2∗IGFBP7 concentrations and the risk of developing AKI [6–9, 11]. In the Topaz study conducted in 420 critically ill adults admitted to 23 participating ICUs, Bihorac et al. showed that urinary TIMP2∗IGFBP7 was an independent factor associated with AKI (KDIGO 2 or 3) within 12 hours [9]. Two different cutoff values were tested: TIMP2∗IGFBP7 > 0.3 (sensitivity of 92% and specificity of 46%) and >2.0 (sensitivity of 37% and specificity of 95%). The median TIMP2∗IGFBP7 in the AKI group was 1.6 (0.2-2.8) versus 0.3 (0.2-0.8) (ng/ml)^2^/1,000 in the non-AKI group. In 50 patients undergoing cardiac surgery, Meersch et al. found that TIMP2∗IGFBP7 > 0.3 (ng/ml)^2^/1,000, which was associated with the diagnosis of AKI (KDIGO≥1) (sensitivity of 80% and specificity of 83%) 4 hours after cardiac surgery [11]. The median TIMP2∗IGFBP7 4 hours after surgery in the AKI group was 1.5 versus 0.2 (ng/ml)^2^/1,000 in the non-AKI group. The median urinary TIMP2∗IGFBP7 concentrations observed in our study were particularly high compared to the values reported in these previous two studies (Table 1). However, these previous studies included unselected ICU patients (only 20 to 30% of whom presented sepsis) and excluded patients with KDIGO 2 or 3 AKI. The present study only included patients with septic shock who had already developed AKI (KDIGO 1 or 2), accounting for the more severe kidney injury and the relatively high urinary TIMP2∗IGFBP7 concentrations observed. Our study is the first to specifically look at the progression from mild and moderate to severe AKI whereas others looked at none or mild AKI progression to moderate or severe AKI [9–12]. Only one study focused on the specific condition of septic shock [12]. Because inflammation is associated with the cellular expression of TIMP2 and IGFBP7 [15], it was also important to confirm the interest of TIMP2∗IGFBP7 in this setting of septic patients (one of the leading causes of AKI in ICU).

Although urinary TIMP2∗IGFBP7 concentrations appear to be an independent factor associated with the onset of severe AKI, our results show that this diagnostic value is clinically relevant only at H24 and not at H72. We hypothesized that the risk of developing KDIGO 3 AKI was related to the early urinary TIMP2∗IGFBP7 concentration reflecting the severity of the initial kidney aggression. The relationship between the initial injury and subsequent diagnostics of severe AKI probably changes with time due to additional kidney injuries (for example, the prescription of nephrotoxic drugs) or tubular cell repair. Urinary TIMP2∗IGFBP7 concentrations are early markers of tubular injury and reflect the short-term risk of severe AKI diagnostics. In contrast, serum creatinine and urine output are known to be late markers of kidney function [16]. The early tubular injury detected by the release of TIMP2∗IGFBP7 would be associated with the worsening of the kidney function. As we show here, TIMP2∗IGFBP7 seems to be poorly associated with kidney function later than 24 hours. TIMP2∗IGFBP7 may be considered only as a short-term reflection of the kidney function.

The results of this study can help to rapidly stratify the risk of progression to KDIGO 3 AKI over the next 24 hours, which could have a number of applications, particularly in clinical research. Several ongoing studies are trying to limit the development of AKI in populations at high risk of AKI. For example, nicotinamide and cellular immunotherapy are currently under study with regard to decreasing the development of AKI following cardiac surgery or in the presence of septic shock. Future clinical studies may also focus on progression to severe AKI, and our results may help investigators to select this population at highest risk of severe AKI. The treatments currently under investigation to avoid the development of AKI could then be tested to avoid further deterioration of kidney function and to promote recovery of kidney function in septic patients.

Our results should not be interpreted as a potential indication for the early initiation of RRT in this population of patients at high risk of progression to KDIGO 3, as two multicenter randomized controlled trials have shown that KDIGO 3 per se does not constitute an indication for the initiation of RRT, which depends on either an urgent indication (hyperkalemia, pulmonary edema, or severe acidosis) or prolonged anuria (>72 h) (AKIKI and IDEAL ICU). Therefore, only some of the patients identified to be at high risk of progression to KDIGO 3 finally required RRT, as in our population, TIMP2∗IGFBP7 was not associated with the need for RRT and only a small proportion of patients who progressed to KDIGO 3 finally required RRT (16/45 KDIGO 3 at H24).

Our study presents several limits. We did not record the evolution of the urine output, creatinine, or TIMP2∗IGFBP7 12 hours after inclusion which could be more sensitive to identifying high-risk patients of KDIGO 3. Only 45 patients were KDIGO 3 at H24 and 47 patients at H72 while we included, respectively, 6 and 7 variables in the logistic regression models. The individual values of TIMP2 and IGFBP7 could not be analyzed separately because the Astute 140™ Meter only provided the product of both biomarkers. Thus, the exact contribution of each biomarker could not be determined. The high number of variables included in the logistic regression models may have resulted in an overfitted effect. This emphasizes again the importance to confirm the results in a bigger cohort of patients. We used the bone criteria to define the septic shock instead of the new SESPIS 3 definition because the inclusions started before the publication of the SEPSIS3 definition. In 20 patients (10 patients KDIGO<3 H24 and 10 patients KDIGO 3 H24), no history of basal creatinine was available and we used back calculation as recommended by the KDIGO. However, a recent study has shown that back calculation of basal creatinine has moderate agreement with the AKI severity based on the measured basal creatinine, and we may have overestimated the prevalence of AKI [17]. Because it is almost impossible to determine the exact onset of sepsis and AKI in our population, our results cannot be analyzed according to the delay between the onset of AKI and the urinary TIMP2∗IGFBP7 concentrations.

## 5. Conclusion

In conclusion, the urinary TIMP2∗IGFBP7 concentration at the early phase of septic shock is an independent factor to identify the population at high risk of progression from mild and moderate to severe AKI over the next 24 hours but not 72 hours. A TIMP2∗IGFBP7 concentration > 2.0 (ng/ml)^2^/1,000 quadruples the risk of the KDIGO 3 level within 24 hours.

## Figures and Tables

**Table 1:** Clinical characteristics and comparison between patients who did not develop severe AKI (KDIGO<3) and those who developed severe AKI (KDIGO 3) during the 24 hours and 72 hours following inclusion.

Parameter	Global *n* = 112	KDIGO<3 H24 *n* = 67	KDIGO 3 H24 *n* = 45	*p* value	KDIGO<3 H72 *n* = 65	KDIGO 3 or death H72 *n* = 47	*p* value
Age (y)	65 (59-75)	65 (55-76)	64 (60-74)	0.93	65 (62-70)	65 (62-71)	0.85
Male (*n* (%))	67 (60)	41 (61)	26 (58)	0.87	40 (61)	27 (57)	0.70
SAPS II	57 (43-70)	55 (42-64)	64 (51-76)	0.01	52 (47-57)	67 (61-73)	0.001
BMI (kg/m^2^)	27 (24-32)	28 (24-33)	26 (22-30)	0.21	28 (25-33)	26 (23-33)	0.56
Basal creatinine (*μ*mol/l)	71 (60-92)	76 (61-94)	71 (57-87)	0.37	73 (69-80)	71 (66-79)	0.44
Comorbidity (*n* (%))							
Diabetes	46 (41)	30 (45)	16 (35)	0.43	29 (45)	17 (36)	0.44
Hypertension	56 (50)	34 (51)	22 (49)	0.99	33 (51)	23 (49)	0.99
Cardiovascular disease	22 (20)	14 (21)	8 (18)	0.49	13 (20)	9 (19)	0.99
Any malignancy	34 (30)	17 (25)	17 (38)	0.23	16 (25)	18 (38)	0.15
Origin of sepsis (*n* (%))							
Pulmonary	73 (65)	43 (64)	30 (67)	0.88	43 (64)	30 (64)	0.9
Gastrointestinal	13 (12)	8 (12)	5 (11)	1	7 (11)	6 (13)	0.91
Soft tissue	7 (6)	4 (6)	3 (7)	0.9	5 (6)	2 (4)	0.73
Urinary	7 (6)	6 (9)	1 (2)	0.4	6 (9)	1 (2)	0.25
Other	5 (4)	3 (4)	2 (4)	1	4 (4)	1 (2)	0.82
Baseline MAP (mmHg)	73 (66-86)	74 (67-87)	71 (62-82)	0.15	74 (70-82)	71 (66-76)	0.02
Baseline heart rate (bpm)	105 (90-123)	101 (87-121)	110 (93-127)	0.17	104 (97-109)	111 (100-114)	0.20
Baseline norepinephrine (*μ*g/kg/min)	0.36 (0.16-0.87)	0.27 (0.15-0.45)	0.60 (0.30-1.16)	0.001	0.32 (0.20-0.37)	0.50 (0.29-0.87)	0.03
Fluid administered (between initiation of catecholamines and first urine sample) (ml/kg)	16 (7-25)	15 (5-23)	19 (11-28)	0.01	15 (9-17)	20 (14-25)	0.01
Baseline lactate (mmol/l)	2.6 (1.6-4.2)	2.2 (1.6-3.7)	3.0 (2.0-5.4)	0.03	2.1 (1.8-2.5)	3.5 (2.9-4.4)	0.001
Mechanical ventilation (*n* (%))	77 (69)	46 (69)	31 (69)	0.85	42 (65)	35 (74)	0.31
Interval between initiation of catecholamines and first urine sample (hours)	2.0 (1.0-4.0)	2.0 (1.0-4.0)	3.0 (1.0-4.0)	0.24	2.0 (2.0-2.7)	3.0 (2.0-3.0)	0.46
Mortality in ICU (*n* (%))	49 (44)	21 (31)	28 (62)	0.002	18 (28)	31 (66)	0.001
ICU length of stay (days)	6.0 (3-14)	7.0 (4.0-15.7)	5.0 (2.5-12.0)	0.05	8.0 (6.0-10.9)	4.0 (3.0-8.1)	0.09
Renal replacement therapy within 72 hours (*n* (%))	19 (17)	3 (4)	16 (35)	0.001	2 (3)	17 (36)	0.001
Baseline urine output (ml/kg/h)	0.53 (0.24-1.19)	0.73 (0.40-1.47)	0.31 (0.16-0.64)	0.001	0.71 (0.54-0.95)	0.31 (0.20-0.49)	0.001
Baseline creatinine (*μ*mol/l)	123 (91-174)	114 (87-139)	163 (108-247)	0.001	114 (106-131)	139 (122-170)	0.009
Baseline TIMP2∗IGFBP7 ((ng/ml)^2^/1,000)	1.45 (0.58-4.13)	0.93 (0.25-1.59)	3.99 (2.17-9.45)	0.001	1.03 (0.77-1.51)	3.03 (1.81-5.11)	0.001

SAPS II: Simplified Acute Physiology Score 2; MAP: mean arterial pressure; ICU: intensive care unit; H0: time of first urine sample. Basal serum creatinine was defined as the serum creatinine level measured during the 12 months preceding the onset of septic shock. In the absence of a previous serum creatinine assay, basal serum creatinine was estimated according to the KDIGO guideline [3]. All baseline parameters have been recorded at inclusion. Baseline urine output was the volume of urine excreted by hour during the 4 to 6 hours preceding the inclusion.

**Table 2:** Stepwise logistic regression risk models for severe AKI 24 hours (H24) or severe AKI and/or death 72 hours (H72) following inclusion.

AKI KDIGO H24				
Variable	H24 (OR (95% CI))	*p* value	H24 (OR (95% CI))	*p* value

Baseline posology of norepinephrine	4.26 (1.16-15.6)	0.03	4.55 (1.43-14.5)	0.01
Baseline fluid administered	1.02 (0.97-1.06)	0.47	—	—
Baseline lactate	1.02 (0.80-1.29)	0.87	—	—
Baseline creatinine	1.01 (1.01-1.02)	0.001	1.02 (1.01-1.3)	0.001
Baseline urine output	0.19 (0.05-0.77)	0.02	0.19 (0.05-0.74)	0.004
Baseline TIMP2∗IGFBP7	1.42 (1.11-1.83)	0.005	1.42 (1.12-1.81)	0.001
Nagelkerke *R*^2^	0.63		0.64	

AKI and/or death H72				
Variable	H72 (OR (95% CI))	*p* value	H72 (OR (95% CI))	*p* value

Baseline mean arterial pressure	0.97 (0.94-1.01)	0.18	—	—
Baseline posology of norepinephrine	1.93 (0.70-5.32)	0.2	—	—
Baseline fluid administered	1.03 (0.99-1.07)	0.12	—	—
Baseline lactate	1.22 (0.98-1.52)	0.08	—	—
Baseline creatinine	1.01 (1.00-1.02)	0.04	1.01 (0.99-1.01)	0.06
Baseline urine output	0.65 (0.30-1.39)	0.27	—	—
Baseline TIMP2∗IGFBP7	1.21 (1.00-1.45)	0.04	1.31 (1.12-1.53)	0.001
Nagelkerke *R*^2^	0.45		0.29	

## Data Availability

The data used to support the findings of this study are included within the article.

## Abbreviations

AKI: Acute kidney injury
AUC: Area under the curve
ICU: Intensive care unit
IGFBP7: Insulin-like growth factor-binding protein 7
KDIGO: Kidney Disease: Improving Global Outcomes
ROC: Receiver operating characteristic
RRT: Renal replacement therapy
SAPS: Simplified acute physiology score
TIMP2: Tissue inhibitor of metalloproteinase type 2.
